# Rivaroxaban modulates electrical and mechanical characteristics of left atrium

**DOI:** 10.1186/1423-0127-20-17

**Published:** 2013-03-15

**Authors:** Chien-Jung Chang, Yao-Chang Chen, Yung-Kuo Lin, Jen-Hung Huang, Shih-Ann Chen, Yi-Jen Chen

**Affiliations:** 1 Graduate Institute of Clinical Medicine, College of Medicine, Taipei Medical University, Taipei, Taiwan; 2 Division of Cardiology, Tungs’ Taichung Metroharbor Hospital, Taichung, Taiwan; 3 Department of Biomedical Engineering and Institute of Physiology, National Defense Medical Center, Taipei, Taiwan; 4 Division of Cardiovascular Medicine, Department of Internal Medicine, Wan Fang Hospital, Taipei Medical University, 111 Hsin-Lung Road Sec. 3, Taipei 116, Taiwan; 5 Division of Cardiology and Cardiovascular Research Center, Taipei Veterans General Hospital, Taipei, Taiwan

**Keywords:** Rivaroxaban, Atrial fibrillation, Factor Xa, Nitric oxide synthase, Cyclooxygenase

## Abstract

**Background:**

Rivaroxaban reduces stroke in patients with atrial fibrillation (AF). Left atrium (LA) plays a critical role in the pathophysiology of AF. However, the electromechanical effects of rivaroxaban on LA are not clear.

**Results:**

Conventional microelectrodes and a whole-cell patch-clamp were used to record the action potentials (APs) and ionic currents in rabbit LA preparations and isolated single LA cardiomyocytes before and after the administration of rivaroxaban. Rivaroxaban (10, 30, 100, and 300 nM) concentration-dependently reduced LA (*n* = 7) AP durations at 90% repolarization (APD_90_) from 76 ± 2 to 79 ± 3, 67 ± 4 (P < 0.05, vs. control), 59 ± 5, (P < 0.01, vs. control), and 56 ± 4 ms (P < 0.005, vs. control), respectively. Rivaroxaban (10, 30, 100, and 300 nM) concentration-dependently increased the LA (*n* = 7) diastolic tension by 351 ± 69 (P < 0.05, vs. control), 563 ± 136 (P < 0.05, vs. control), 582 ± 119 (P < 0.05, vs. control), and 603 ± 108 mg (P < 0.005, vs. control), respectively, but did not change LA contractility. In the presence of L-NAME (100 μM) and indomethacin (10 μM), additional rivaroxaban (300 nM) treatment did not significantly further increase the LA (*n* = 7) diastolic tension, but shortened the APD_90_ from 73 ± 2 to 60 ± 6 ms (P < 0.05, vs. control). Rivaroxaban (100 nM) increased the L-type calcium current and ultra-rapid delayed rectifier potassium current, but did not change the transient outward potassium current in isolated LA cardiomyocytes.

**Conclusions:**

Rivaroxaban modulates LA electrical and mechanical characteristics with direct ionic current effects.

## Background

Arial fibrillation (AF) is the most common sustained arrhythmia which predisposes to early activation of platelets, induction of coagulation factors, and ultimately formation of thrombi in left atrium (LA), which contributes to thromboembolic morbidity and mortality [1,2]. LA plays a major role in the genesis of AF, and also harbors activated platelets and increased prothrombotic factors [2]. These AF-activated prothrombotic factors, activated factor X (FXa) and downstream thrombin, may exert their electromechanical effects on cardiomyocytes other than thrombosis through activating protease-activated receptors (PARs) [3]. FXa elicits intracellular signaling responses through the activated PAR2 and possibly also through PAR1 in endothelial and smooth muscle cells [4]. Activated PAR1 coupled with G_q_, G_i_, and/or G_12/13_ stimulation mediated nitric oxide (NO) production by NO synthase (NOS) [5]. In various tissues, PAR2 activation caused arachidonic acid release and prostanoid formation which involved cyclooxygenase (COX) induction via multiple signaling pathways [5]. Accordingly, AF-induced FXa which activates PAR1 and PAR2 could modulate LA electrical and mechanical characteristics through NO and prostanoid formation [5,6].

Rivaroxaban selectively binds to the FXa and reduces thrombin formation, which prevents stroke in AF patients [7]. However, it is not clear whether rivaroxaban modulates electrical and mechanical characteristics of the LA. The purposes of this study were to investigate the electrical and mechanical effects of rivaroxaban on LA and evaluate the roles of NOS and COX in the effects of FXa.

## Methods

### Electromechanical and pharmacological studies of the LA preparations

Experiments in this study conformed to the institutional *Guide for the Care and Use of Laboratory Animals.* Male rabbits (weighing 1 ~ 2 kg) were anesthetized with an intraperitoneal injection of sodium pentobarbital (100 mg/kg). A midline thoracotomy was then performed, and the heart and lungs were removed as described previously [8,9]. To dissect the LA, the LA was opened by an incision along the mitral valve annulus, extending from the coronary sinus to the septum, in Tyrode’s solution with a composition (in mM) of 137 NaCl, 4 KCl, 15 NaHCO_3_, 0.5 NaH_2_PO_4_, 0.5 MgCl_2_, 2.7 CaCl_2_, and 11 dextrose. Preparations were connected to a WPI model FD223 electrometer under a tension of 150 mg as described previously [10,11]. One end of the preparations was pinned with needles to the bottom of a tissue bath. The other end was connected to a Grass FT03C force transducer with a silk thread. The adventitial or epicardial side of the preparations faced upwards. The LA tissue strips were superfused at a constant rate (3 ml/min) with Tyrode’s solution saturated with a 97% O_2_-3% CO_2_ gas mixture. The temperature was maintained at 37°C, and the preparations were allowed to equilibrate for 1 h before the electrophysiological assessment.

Transmembrane action potentials (APs) were recorded by machine-pulled glass capillary microelectrodes filled with 3 mol/L of KCl which were connected to a WPI Duo 773 electrometer under a tension of 150 mg. Electrical and mechanical events (contractile force and diastolic tension) were simultaneously displayed on a Gould 4072 oscilloscope and a Gould TA11 recorder. Using a data acquisition system, signals were recorded with DC coupling and a 10-kHz low-pass filter cutoff frequency. Signals were recorded digitally with a 16-bit accuracy at a rate of 125 kHz. Electrical stimulation was provided using a Grass S88 stimulator through a Grass SIU5B stimulus isolation unit. The AP amplitude (APA) was obtained by measuring the difference between the resting membrane potential (RMP) or maximum diastolic potential and the peak of AP depolarization. AP durations (APDs) at repolarization of 90%, 50%, and 20% of the APA were respectively measured as APD_90_, APD_50_, and APD_20_. The RMP, APA, APD_90_, APD_50_, APD_20_, and contractile forces were measured under 2-Hz pacing of the LA before and after the sequential administration of rivaroxaban (10, 30, 100, and 300 nM). In order to study the mechanical effects of rivaroxaban, an N^G^-nitro-L-arginine methyl ester (L-NAME) solution (an NOS inhibitor, 100 μM) and indomethacin (a non-selective COX inhibitor, 10 μM) solution and rivaroxaban solution (300 nM) were sequentially added to LA preparations.

### Isolation of LA cardiomyocytes and a whole-cell patch-clamp

Single LA myocytes were enzymatically dissociated through the same procedure as described previously [12]. A whole-cell perforated with amphotericin B of 300 μg/ml (for L-type calcium current, *I*_Ca-L_) or ruptured (for others) patch clamp was performed in single isolated cardiomyocyte before and after the administration of rivaroxaban (100 nM) using an Axopatch 1D amplifier (Axon Instruments, Foster City, CA, USA) at 35 ± 1°C[12]. Borosilicate glass electrodes (o.d., 1.8 mm) with tip resistances of 3 ~ 5 MΩ were used. Before the formation of the membrane-pipette seal, the tip potentials were zeroed in Tyrode’s solution. The junction potentials between the bath and pipette solution (9 mV) were corrected for the AP recordings. APs were recorded in the current-clamp mode, and the ionic currents were recorded in the voltage-clamp mode. A small hyperpolarizing step from a holding potential of −50 mV to a test potential of −55 mV for 80 ms was delivered at the beginning of each experiment. The area under the capacitative currents was divided by the applied voltage step to obtain the total cell capacitance. Normally 60% ~ 80% series resistance (Rs) was electronically compensated for. APs were elicited in cells from the LA through brief current pulses at 1 Hz. The extracellular solution contained the basic composition (in mM) of NaCl 137, KCl 5.4, HEPES 10, MgCl_2_ 0.5, CaCl_2_ 1.8, and glucose 10. The solution was titrated to a pH of 7.4 with NaOH. Micropipettes were filled with a solution containing (in mM) CsCl 130, MgCl_2_ 1, Mg_2_ATP 5, HEPES 10, EGTA 10, NaGTP 0.1, and Na_2_ phosphocreatine 5, (adjusted to a pH of 7.2 with CsOH) for the *I*_Ca-L_ and containing (in mM) KCl 20, K aspartate 110, MgCl_2_ 1, Mg_2_ATP 5, HEPES 10, EGTA 0.5, LiGTP 0.1, and Na_2_ phosphocreatine 5, (adjusted to a pH of 7.2 with KOH) for the AP and potassium currents. The *I*_Ca-L_ was measured as an inward current during depolarization from a holding potential of −50 mV to testing potentials ranging from −40 to +60 mV in 10-mV steps for 300 ms at a frequency of 0.1 Hz. The NaCl and KCl in the external solution were respectively replaced by TEACl and CsCl. The transient outward potassium current (*I*_to_) was studied with a double-pulse protocol. A 30-ms pre-pulse from −80 to −40 mV was used to inactivate the sodium channels, followed by a 300-ms test pulse to +60 mV in 10-mV steps at a frequency of 0.1 Hz. CdCl_2_ (200 μM) was added to the bath solution to inhibit the *I*_Ca-L_. The *I*_to_ was measured as the difference between the peak outward current and steady-state current [13]. The ultra-rapid delayed rectifier potassium current (*I*_Kur_) was studied with a double-pulse protocol, consisting of a 100-ms depolarizing pre-pulse to +40 mV from a holding potential of −50 mV, followed by 150-ms voltage steps from −40 to +60 mV in 10-mV increments at room temperature to provide an adequate temporal resolution. The *I*_Kur_ was measured as 4-aminopyridine (1 mM)-sensitive currents [14,15].

### Statistical analysis

All continuous variables are expressed as the mean ± standard error of the mean (SEM). A one-way repeated-measures analysis of variance (ANOVA) was used to compare the difference before and after drug administration on LA. The electrophysiological and mechanical characteristics between different groups were compared by a Wilcoxon rank-sum test or unpaired *t*-test depending on the outcome of the normality test. A P value of less than 0.05 was considered statistically significant.

## Results

### Rivaroxaban affects the electric and mechanical characteristics of LA

**Figure 1:**
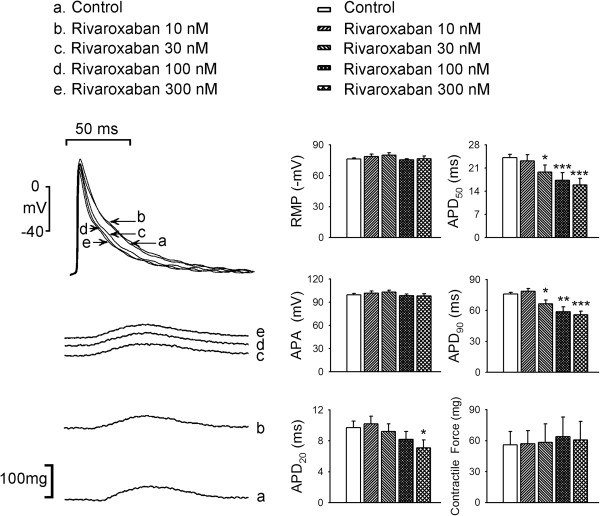
**Effects of rivaroxaban on the left atrial (LA) action potential (AP) and contractility.** An example and average data (*n* = 7) show that rivaroxaban (10, 30, 100, and 300 nM) concentration-dependently shortened AP durations (APD_20_, APD_50_, and APD_90_) of LA. The resting membrane potential (RMP), AP amplitude (APA), and contractile forces of LA did not significantly change with rivaroxaban treatment. * P < 0.05, ** P < 0.01, *** P < 0.005 vs. control.

**Figure 2:**
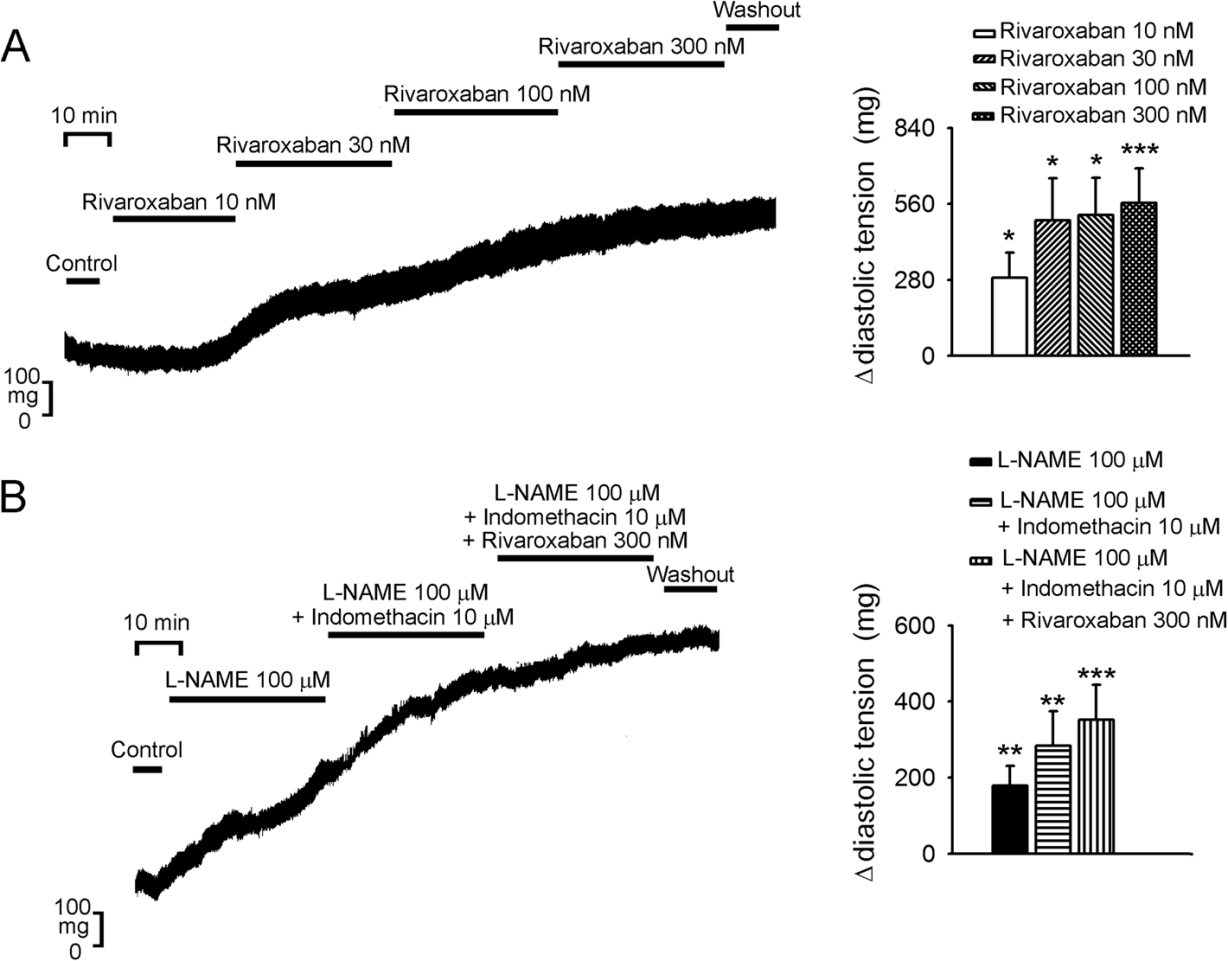
**Effect of rivaroxaban with and without N**^**G**^**-nitro-L-arginine methyl ester (L-NAME) or indomethacin on the left atrial (LA) diastolic tension.** (**A**) An example and average data (*n* = 7) show that rivaroxaban concentration-dependently increased the diastolic tension of LA. (Δ, change in the diastolic tension) (**B**) An example and average data (*n* = 7) show that L-NAME, indomethacin, and additional rivaroxaban sequentially increased the diastolic tension of the LA. * P < 0.05, ** P < 0.01, *** P < 0.005 vs. control.

**Figure 3:**
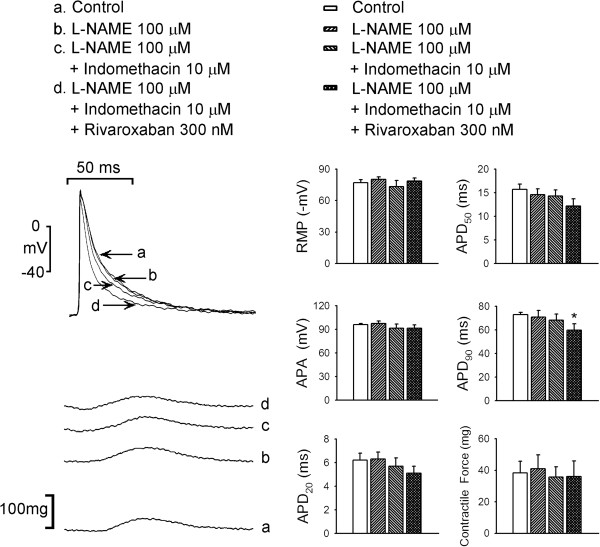
**Effects of rivaroxaban (300 nM) with and without N**^**G**^**-nitro-L-arginine methyl ester (L-NAME, 100 μM), or indomethacin (10 μM) and on the left atrial (LA) action potential (AP) and contractility.** An example and average data (*n* = 7) show that rivaroxaban (10, 30, 100, and 300 nM) solutions concentration-dependently shortened the 90% repolarization of the AP duration (APD_90_) of LA. The resting membrane potential (RMP), AP amplitude (APA), and contractile forces of LA did not significantly change with L-NAME, indomethacin and additional rivaroxaban treatment. * P < 0.05 vs. control.

As shown in Figure 1, rivaroxaban (10, 30, 100, and 300 nM) concentration-dependently reduced the APD_20_, APD_50_, and APD_90_ in LA. However, rivaroxaban did not significantly affect the RMP, APA, or contractile force of LA. Moreover, rivaroxaban (10, 30, 100, and 300 nM) concentration-dependently increased LA diastolic tension, which could not be reversed after washout for 30 min (Figure 2A). The treatment with L-NAME (100 μM), add-on indomethacin (10 μM), and additional rivaroxaban (300 nM) increased the LA diastolic tension as compared to control, respectively (Figure 2B), but did not affect contractile forces of LA (Figure 3). In addition, the increment in diastolic tension by L-NAME was less than that in the LA treated with 30 nM rivaroxaban (P < 0.05), 100 nM rivaroxaban (P < 0.05), and 300 nM rivaroxaban (P < 0.05). In the presence of L-NAME, indomethacin administration (10 μM) further increased the LA diastolic tension which was comparable to that of LA treated with rivaroxaban (30, 100, and 300 nM). In the presence of L-NAME and indomethacin, the additional rivaroxaban (300 nM) treatment did not further significantly increase the LA diastolic tension, and the overall increment in the LA diastolic tension after treatment of rivaroxaban (300 nM) did not differ in the presence or absence of L-NAME and indomethacin.

As shown in Figure 3, in the presence of L-NAME (100 μM) and indomethacin (10 μM), additional treatment with rivaroxaban (300 nM) significantly shortened the APD_90_ but did not affect the RMP or APA of LA.

### Effects of rivaroxaban on AP configurations in LA myocytes

**Figure 4:**
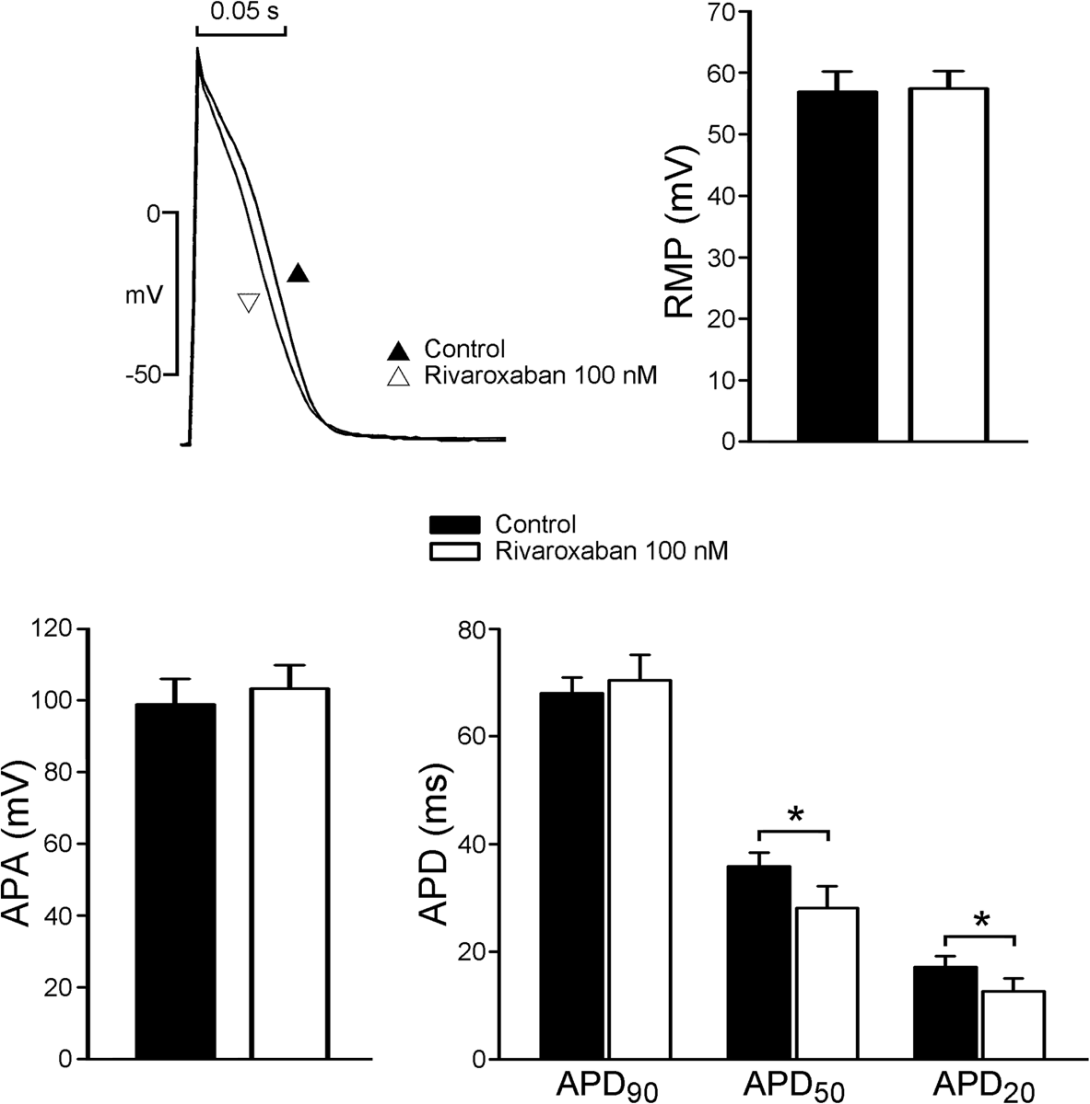
**Effects of rivaroxaban on isolated left atrial (LA) myocytes.** An example and average data show that rivaroxaban shortened the action potential durations (APD_20_ and APD_50_) of LA (*n* = 7). * P < 0.05 vs. control.

Rivaroxaban had rapid effects on the electrical activity of LA myocytes and reached a steady state in 3 ~ 5 min after its superfusion. Figure 4 presents examples of the AP morphology of LA myocytes before and after rivaroxaban (100 nM) administration (*n* = 7). Superfusion with rivaroxaban significantly reduced APD_50_ and APD_20_ in LA myocytes. However, rivaroxaban (100 nM) did not affect the APA or RMP in LA myocytes.

### Effect of rivaroxaban on membrane currents of LA myocytes

**Figure 5:**
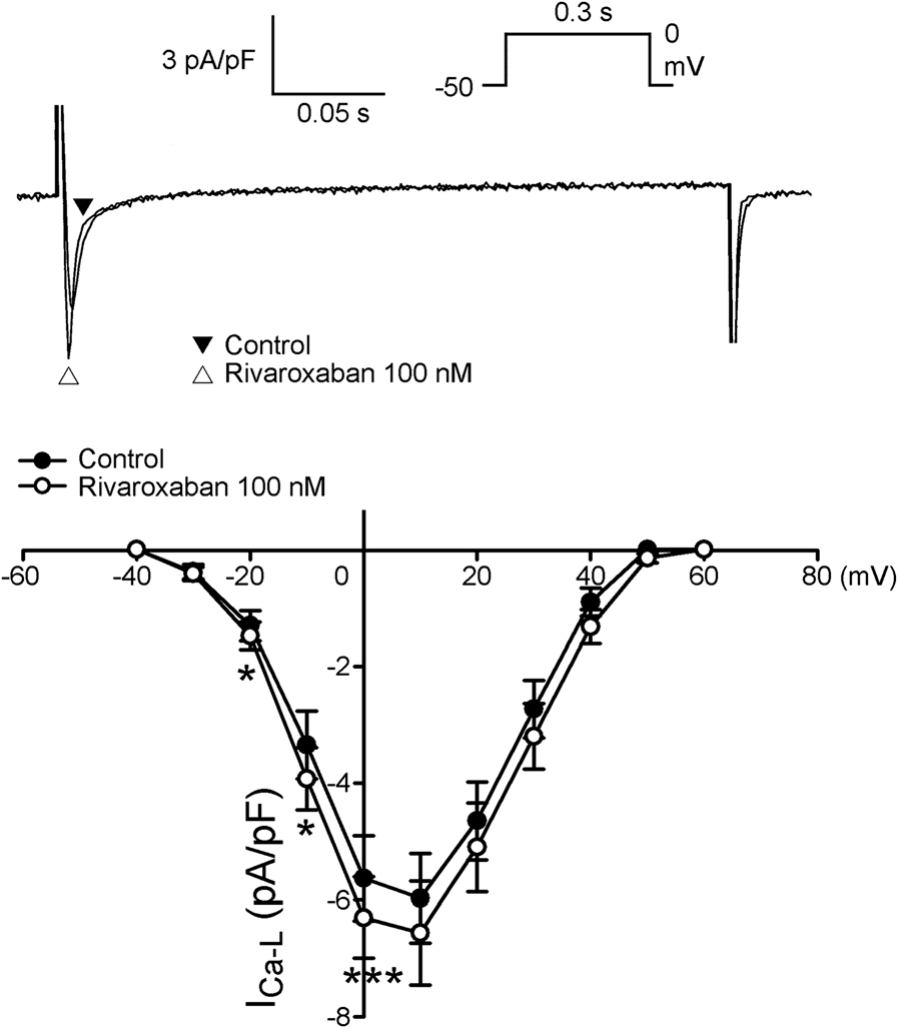
**Effect of rivaroxaban on L-type calcium current (*I***_**Ca-L**_**) of left atrial (LA) myocytes.** An example and average data show the I-V relationship of the *I*_Ca-L_ before and after rivaroxaban (100 nM) administration in LA myocytes (*n* = 11). * P < 0.05 vs. control. The inset shows the clamp protocol.

**Figure 6:**
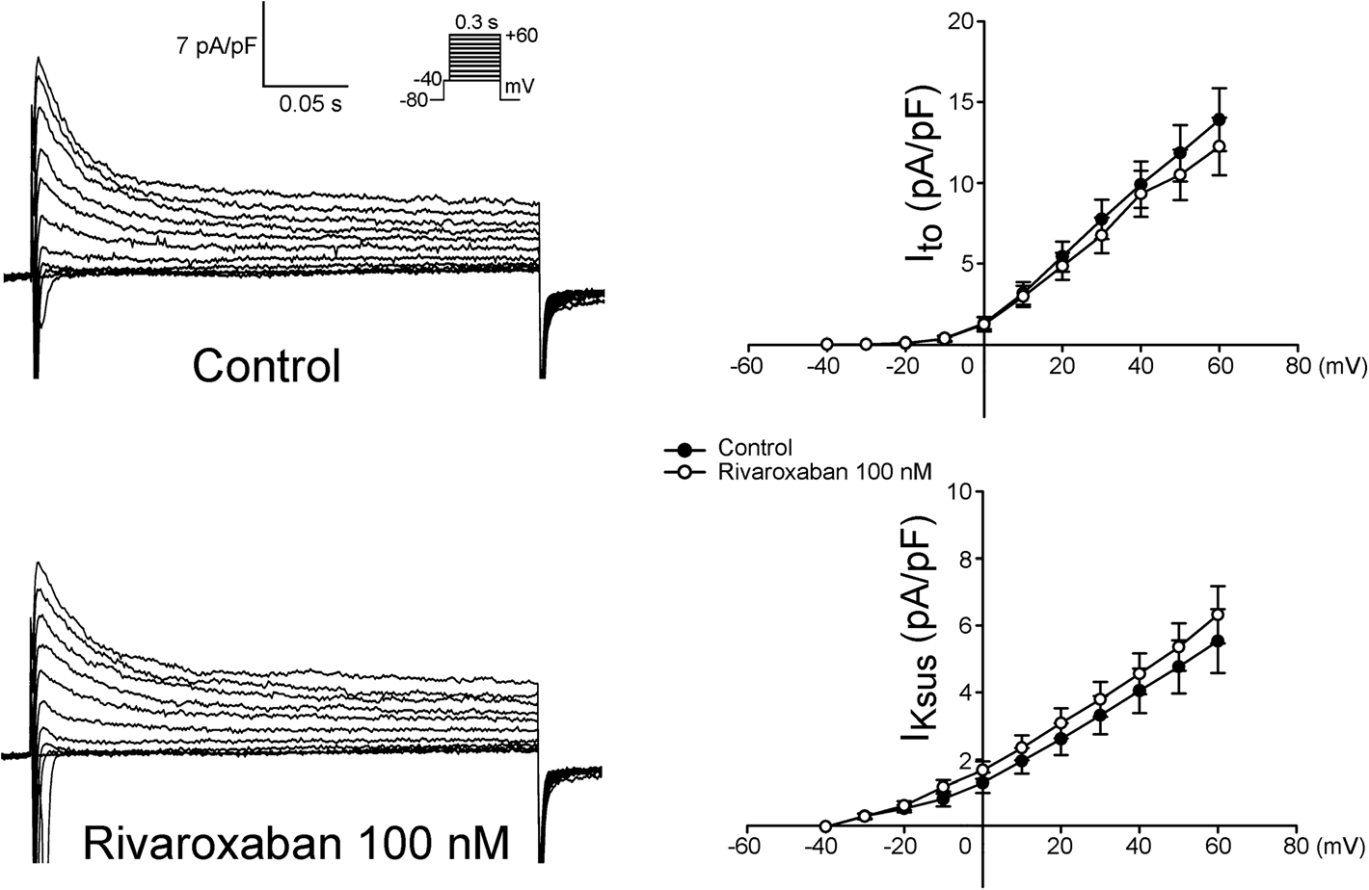
**Effects of rivaroxaban on the transient outward current (*I***_**to**_**) of left atrial (LA) myocytes.** An example and average data show the I-V relationship of the *I*_to_ before and after rivaroxaban administration in LA myocytes (*n* = 12). The inset shows the clamp protocol.

**Figure 7:**
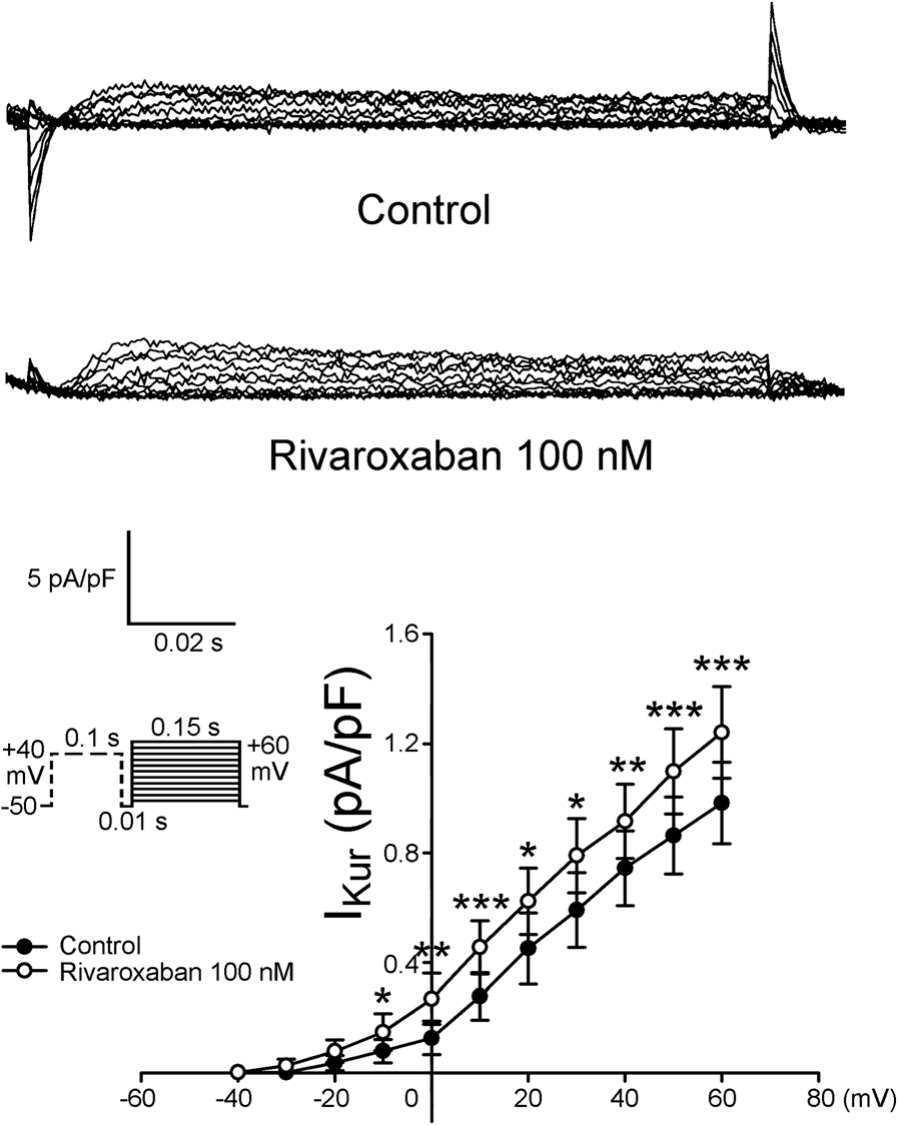
**Effects of rivaroxaban on the ultra-rapid delayed rectifier potassium current (*I***_**Kur**_**) of left atrial (LA) cardiomyocytes.** This figure shows a tracing and the I-V relationship of the *I*_Kur_ before and after rivaroxaban (100 nM) administration in LA myocytes (*n* = 12), *P < 0.05 vs. control. The inset shows the clamp protocol.

Figure 5 shows tracings and the I-V relationship of the *I*_Ca-L_ before and after rivaroxaban (100 nM) administration in LA myocytes, and it was found that rivaroxaban increased the peak *I*_Ca-L_ by 11.8% (elicited from −40 mV to +60 mV, *n* = 11). However, as shown in Figure 6, rivaroxaban (100 nM) did not significantly change the *I*_to_. Moreover, Figure 7 shows that rivaroxaban (100 nM) significantly increased the peak *I*_Kur_ by 29.6% (elicited from −40 mV to +60 mV, *n* = 12).

## Discussion

To reduce thromboembolic complications with AF, several new antithrombotic agents were developed with non-inferior antithrombotic effects and fewer hemorrhagic complications than the traditional vitamin K antagonist [16,17]. It’s well known that AF-activated prothrombotic factors, activated factor X (FXa) and downstream thrombin, exert their electromechanical effects on cardiomyocytes other than thrombosis through activating protease-activated receptors (PARs) [3]. Accordingly, the reduction in FXa may also modulate electrical and mechanical characteristics of the LA by inhibiting NO and prostanoid [4,5]. This study attempted to investigate the electrical and mechanical effects of rivaroxaban on LA at comparable physiological doses and also evaluated the inhibitors of NOS and COX. Rivaroxaban reversibly bound to the FXa and diminished PAR1 and PAR2 activation. These two activated G-protein-coupled receptors promote the production of prostanoid and NO which induce vascular dilatation and reduce wall tension. In this study, rivaroxaban administration resulted in concentration-dependent shortening of APDs (Figure 1) and an increasing wall tension of LA (Figure 3). Moreover, in Figures 1 and 3, the diastolic tension increased earlier (with 10 nM rivaroxaban) than the occurrence of APD shortening (with 30 nM rivaroxaban). The earlier increased wall tension may also have contributed to the shortening of the AP through mechanoelectrical feedback [18-20], in addition to the direct effects of rivaroxaban on LA cardiomyocytes. According to a previous study, stretching of myocardiocytes shortened the APD, which implies a possible mechanism of electric modulation by rivaroxaban [21]. A maximal plasma concentration (C_max_), of around 125 μg/L or 300 nM, of rivaroxaban was reached within 2 ~ 4 h after oral administration after a single oral dose in healthy volunteers [22,23]. Therefore, the concentrations used in this experiment are clinically relevant. However, there is no report available so far to show that rivaroxaban administered at clinically relevant dose increases atrial diastolic pressure. Moreover, this study did not evaluate the effects of rivaroxaban (300 nM) alone or L-NAME with rivaroxaban on LA.

In this study, we found that L-NAME significantly increased the LA wall tension but this effect is less than that by rivaroxaban. This finding implied that blockage of NOS mediated a portion of the effects of rivaroxaban on LA to increase the wall tension. Additional administration of indomethacin, which blocked COX and reduced prostanoid production, further elevated the wall tension of LA to a level comparable to that induced by rivaroxaban. In the presence of L-NAME and indomethacin, the additional administration of rivaroxaban (300 nM) did not significantly increase the wall tension, but the total increment in the wall tension was comparable to the increase by rivaroxaban (300 nM) as shown in Figure 2. The blockage of the FXa by rivaroxaban reduced the production of NO and prostanoid, which coordinately increased the wall tension of LA. The blockage of NOS by L-NAME represented a partial mechanical effect, while the additional blockage of COX by indomethacin seemed to nearly complete the mechanical effect of rivaroxaban on LA. However, the sequential addition of L-NAME and indomethacin did not significantly change the APDs, but additional rivaroxaban (300 nM) treatment shortened the APD_90_ compared to the baseline (Figure 2). It is not clear whether this electrical effect of rivaroxaban on LA is directly mediated by rivaroxaban or resulted from diminished activation of PARs. Besides, we also didn’t use the NOS and COX activator to elucidate the mechanisms of rivaroxaban-increased diastolic tension in LA or their direct AP and ionic effects in isolated LA myocytes.

In experiments on isolated single myocytes, we found that rivaroxaban (100 nM) shortened the APD_20_ and APD_50_, but did not affect the APD_90_, RMP, or APA. The less shortening of the APD in isolated single cardiomyocyte by rivaroxaban suggested that the greater shortening of APD in LA tissue preparations may have been caused by mechanoelectrical feedback due to the increased wall tension with stretched myocytes in tissue preparation experiments. The ionic current experiments showed that rivaroxaban did not change the *I*_to_ but significantly increased the *I*_Kur_, which may have contributed to its effect in shortening the APD. Rivaroxaban also increased the *I*_Ca-L_, which may have been a result of repolarization homeostasis and implies that the increasing LA wall tension was due to intracellular calcium loading. These findings suggest that rivaroxaban at clinically relevant concentrations directly affects ionic currents in LA myocytes. In order to correlate the clinical settings, we use 100 nM of rivaroxaban to measure its ionic current effects, which are closely related to the known plasma concentrations in humans [24]. However, it is not clear whether the higher concentration of rivaroxaban (300 nM) may have stronger ionic effects. Moreover, we did not use purified FX in the tissue preparation experiments to elucidate its potential competition effects to rivaroxaban.

## Conclusions

Rivaroxaban modulates LA electrical characteristics with direct effects on ionic currents, and regulates mechanical characteristics, which can be attenuated by the inhibitions on NOS and COX.

## Competing interests

The authors declare that they have no competing interests.

## Authors’ contributions

CJC and YKL interpreted the data and drafted the manuscript. YCC and JHH performed the experiments and revised it for scientific content. SAC and YJC conceived of this study, and participated in its design and coordination. All authors read and approved the final manuscript.
